# Impact of artificial intelligence-driven urban renewal strategies green economic efficiency and resident health in China

**DOI:** 10.3389/fpubh.2025.1577511

**Published:** 2025-08-01

**Authors:** Jing Peng, Xin Fu, Yufan Peng, Yang Ding

**Affiliations:** ^1^College of Art and Design, Wuhan Textile University, Wuhan, China; ^2^Hubei Province Changjiang Industry Investment Group Co., Ltd., Wuhan, China

**Keywords:** smart cities, urban renewal strategies, green economic efficiency, resident health, green total factor productivity

## Abstract

**Introduction:**

This study investigates the impact of AI-driven smart city policies on green economic efficiency and public health. It further explores how industrial structure rationalization, upgrading, and technological innovation capacity moderate these effects, aiming to provide actionable insights for sustainable urban governance.

**Methods:**

To account for potential policy spillover effects, the study adopts a Difference-in-Differences (DID) approach integrated with a Spatial Durbin Model (SDM). The analysis incorporates AI-enabled smart city renewal strategies into the empirical framework, focusing on their influence on green economic efficiency and public health across varying levels of industrial structure and innovation capacity. Data were sourced from the World Health Organization Global Health Observatory.

**Results:**

Empirical findings demonstrate that AI-driven smart city strategies significantly enhance green economic efficiency (coefficient = 0.098, *p* < 0.01) and public health outcomes (coefficient = 0.085, *p* < 0.01). The positive effects are amplified by rationalized and upgraded industrial structures. Notably, the gains in green economic efficiency are more substantial in regions with lower technological innovation capacity, while regions with higher innovation capacity benefit more in terms of improved public health.

**Discussion:**

The results underscore the strategic importance of aligning AI applications with industrial and innovation policy to foster sustainable urban development. Policymakers are encouraged to leverage AI in optimizing industrial structures, promoting green growth, and integrating health policy with technological innovation to improve urban residents’ quality of life.

## 1 Introduction

With the rapid acceleration of global urbanization, cities are increasingly facing complex challenges related to escalating resource consumption, worsening environmental pollution, and growing public health concerns (1, 2). In response to these pressing issues, many countries have initiated the development of smart cities, which leverage advanced digital technologies and artificial intelligence (AI)-driven management systems to optimize resource allocation, improve environmental quality, and enhance residents’ overall quality of life. At the heart of smart cities lies the integration of AI, the Internet of Things (IoT), and big data, which collectively elevate urban management to unprecedented levels of intelligence. This enables cities to achieve critical objectives, such as fostering a green economy and promoting sustainable development (3, 4). The application of AI in smart cities demonstrates remarkable potential, particularly in advancing green economic efficiency and improving public health outcomes. Through the deployment of intelligent decision-support systems, smart cities can manage energy consumption more effectively, minimize carbon emissions, and address public health challenges more proactively (5). These systems not only optimize resource use but also strengthen urban resilience, thereby providing robust and dynamic support for realizing green economic objectives. The effectiveness of smart city renewal strategies varies significantly across different socioeconomic contexts. Economically developed regions typically benefit from advanced digital infrastructure (6), higher technological adoption capacity, and greater financial resources, enabling them to leverage smart city initiatives more effectively to drive green economic development and enhance public health. In contrast, economically underdeveloped areas, constrained by limited technological resources and weaker policy implementation, may struggle to fully harness the potential of AI. Moreover, the digital divide in these regions could exacerbate social inequality, further widening disparities in access to smart city benefits. City size also plays a crucial role in the application of AI-driven smart city renewal. Large cities, characterized by high data density and strong policy support, are more likely to maximize the potential of AI technologies. In contrast, small and medium-sized cities may encounter greater implementation challenges due to resource constraints and lower technological adoption rates. Additionally, while smart city renewal strategies enhance healthcare accessibility and optimize public health management, they may also contribute to health disparities. Low-income groups, the older adult, and individuals with lower educational attainment may face barriers to accessing the benefits of smart healthcare and green mobility due to economic constraints, digital literacy gaps, and insufficient social support. By aligning technological innovation with environmental and social goals, smart cities significantly contribute to enhancing the well-being of urban populations and ensuring a more sustainable future.

In the existing literature, numerous studies have explored the impact of smart city development on green economic efficiency and residents’ health. Regarding green economic efficiency, Liu et al. (7) analyzed data from 279 Chinese cities between 2008 and 2020 to investigate the role of smart city development in promoting urban green economies. Their findings indicate that smart cities contribute to reducing pollution and energy consumption, with this impact varying across different regions. Qian et al. (8) examined the role of smart city development in fostering green economic growth in China, revealing that smart cities promoted green economic growth by driving economic development, reducing energy consumption, and decreasing waste emissions, particularly in large cities and non-resource-based cities. In terms of residents’ health, Wang and Zhou (9) explored the impact of smart city investment on subjective life quality, finding that information and communication technology had a negative effect on life satisfaction and well-being, while human capital had a positive effect. The impact of smart city investments varied significantly across different age groups and education levels. Liu et al. (10) highlighted the role of government information infrastructure in smart cities in alleviating information asymmetry between businesses and the government, which clarified the boundary conditions of AI in the relationship between e-government development and environmental pollution by enterprises, thereby improving public health and safety performance in urban areas. These studies demonstrate that most existing research focuses on improving green economic efficiency, with relatively limited attention given to how smart city renewal strategies can enhance residents’ health. Urban renewal strategies encompass not only the improvement of the ecological environment but also the enhancement of residents’ health, a dimension that remains underexplored in current smart city research. This study aims to address this gap by examining how AI-driven smart city renewal strategies affect both green economic efficiency and residents’ health.

Against the backdrop of accelerating global urbanization and digitalization, smart city development has become a critical tool for enhancing urban governance, optimizing resource allocation, and promoting sustainable development. However, current urban renewal policies primarily emphasize infrastructure development and industrial upgrading, while the impact of smart city renewal on public health remains relatively underexplored. Examining how AI-driven urban renewal strategies influence green economic efficiency and public health not only contributes to refining smart city policies but also provides a scientific foundation for governments in formulating health equity policies. Accordingly, this study centers on several key research questions. First, how do AI-driven smart urban renewal strategies influence green economic efficiency and public health? Specifically, the investigation aims to assess the role of AI technologies in smart urban renewal processes, with a particular focus on their contributions to optimizing the urban environment, improving resource allocation efficiency, fostering green economic development, and enhancing residents’ health outcomes. Second, how do rationalized industrial structure, industrial upgrading, and technological innovation capacity moderate the effectiveness of smart urban renewal strategies? This dimension of the study seeks to examine how variations in industrial structure and the capacity for technological innovation affect the outcomes of smart urban renewal initiatives, thereby influencing both green economic efficiency and public health. Finally, the study investigates how regional heterogeneity in technological innovation capacity and industrial structure characteristics results in differentiated impacts of smart urban renewal strategies across various regions.

The contributions and innovations of this study, developed in response to the aforementioned research questions, are reflected in the following key aspects:

1. This study integrates AI technologies with smart urban renewal strategies to examine their potential in promoting green economic efficiency and public health. By constructing a comprehensive empirical model, it systematically analyzes the impact of AI-driven urban renewal initiatives on these critical dimensions.
2. Several moderating variables—namely, rationalization of structural patterns (RSP), industrial upgrading, and technological innovation capacity—are introduced to address a gap in the existing literature regarding their influence on the effectiveness of smart urban renewal policies. The inclusion of these variables facilitates a more refined analysis of policy performance across varying economic contexts.
3. A Difference-in-Differences (DID) model is employed to estimate the causal effects of smart urban renewal policies. Additionally, a Spatial Durbin Model (SDM) is utilized to control for potential policy spillover effects, thereby enhancing the robustness of the empirical findings. This methodological framework improves the ability to account for external confounding factors encountered during policy implementation, resulting in more reliable conclusions.
4. The study further explores how regional disparities in technological innovation capacity and industrial structure influence the outcomes of smart urban renewal strategies. This analysis provides policy-makers with targeted insights to support the design of region-specific interventions and optimize policy effectiveness.

Through these contributions, the study not only advances the theoretical understanding of the intersection between AI technologies and smart urban renewal but also offers empirical evidence to support the formulation of effective and context-sensitive policy measures.

## 2 Literature review

### 2.1 The relationship between smart cities and green economic efficiency

Smart cities refer to urban systems that utilize information technologies—particularly advanced technologies such as AI and the IoT—to improve the efficiency of infrastructure management, optimize resource allocation, and promote the development of a green economy (11, 12). According to smart city governance theory, the deep integration of advanced technologies like AI into urban governance systems not only enhances responsiveness in domains such as transportation, energy, and the environment but also establishes more flexible and precise mechanisms for resource allocation. This lays a solid foundation for the transition toward a green economy. Meanwhile, green growth theory provides theoretical support for examining how smart urban renewal contributes to improvements in green efficiency. Green growth emphasizes expanding economic output without increasing environmental burdens. In this context, the empowering role of AI in smart urban renewal aligns with the dual objectives of improving efficiency and ensuring environmental sustainability.

In recent years, amid increasing global attention to the Sustainable Development Goals, the development of smart cities has emerged as a critical pathway for driving green economic development (13). Smart cities not only pursue efficiency and innovation in economic development but also emphasize environmental protection and the sustainable use of resources. Li et al. (14) identified six core dimensions of smart cities—economic, environmental, transport, governance, among others—which, when optimized, contribute to improved green economic efficiency. Further empirical evidence from Qian et al. (15) demonstrated that the application of AI in smart cities can promote green economic growth by enhancing energy management, pollution control, and resource utilization efficiency. Moreover, the green development of smart cities involves approaches such as the intelligent management of transportation systems and the enhancement of building energy efficiency (16). Nevertheless, existing studies often focus on isolated dimensions of smart city development and tend to overlook the multidimensional impacts of differing urban characteristics and stages of development on green economic efficiency.

### 2.2 The impact of AI on public health

Public health is one of the core objectives in the development of smart cities, and the role of AI in enhancing residents’ health has increasingly garnered academic attention (17). In recent years, AI has made substantial progress in the healthcare sector, particularly in areas such as disease prediction, intelligent diagnostics, and personalized health management (18). For instance, AI can leverage big data analytics to forecast infectious disease outbreaks and optimize resource allocation, thereby improving the efficiency of public health management (19). However, the influence of AI on public health within smart cities extends beyond the healthcare domain. It also encompasses the intelligent optimization of the urban environment, including air quality monitoring, noise control, and traffic management. These environmental factors collectively affect overall health outcomes (20). Wang and Zhou (9) argued that intelligent urban environments can enhance quality of life, reduce environmental pollution, and improve accessibility to healthcare services, thereby contributing to better public health outcomes. Despite these insights, existing research on how AI optimizes the relationship between the urban environment and public health remains limited. In particular, there is a notable lack of empirical studies that offer a comprehensive analysis of these interactions.

### 2.3 Industrial structure optimization and smart city development

The optimization of industrial structure plays a critical role in the sustainable development of smart cities (21). An optimized industrial structure not only enhances economic efficiency but also reduces environmental pollution and improves resource utilization, thereby contributing to the advancement of a green economy (22). The theory of industrial upgrading provides a theoretical foundation for this process. According to this theory, the evolution of industrial structure is driven by both policy initiatives and technological advancement. In particular, intelligent technologies represented by AI have accelerated the transition toward high value-added and low-carbon industries. The development of smart cities requires not only the growth of high-tech industries but also the transformation and upgrading of traditional sectors—an endeavor that is often closely tied to technological innovation. Gaska and Generowicz (23) highlight a significant positive correlation between industrial structure optimization and technological innovation capacity, noting that innovation can drive structural adjustments that foster green development and improvements in public health within smart cities. The rationalization and upgrading of industrial structure can facilitate high-quality economic development while simultaneously enhancing environmental outcomes (24). Although previous studies have examined the relationship between industrial structure and smart city construction, systematic research on the specific effects of industrial structure optimization on green economic efficiency and public health within the context of smart cities remains limited.

### 2.4 Policy spillover effects and spatial modeling

Policy spillover effects represent a critical consideration in the study of smart city policies (25). Due to the interconnectivity and interactions among cities, the impacts of such policies are often not confined to the regions where they are implemented but may extend to neighboring areas. The DID model and the SDM are two widely adopted approaches for evaluating policy spillover effects (26). The DID model effectively estimates the true causal effects of policy interventions by controlling for heterogeneity across time and space. For example, Wang et al. (27) demonstrate that in the context of smart city initiatives, the DID model can capture the changes before and after policy implementation, thereby revealing the underlying effects of the policy. In contrast, the SDM accounts for spatial dependencies and spillover effects across geographic regions, making it particularly suitable for identifying interactive influences among cities. As illustrated by Zhang and Wen (28), the SDM enhances the precision of empirical analysis by incorporating spatial interactions into the estimation process. However, existing studies tend to focus on the policy impacts within single cities and often overlook the broader spatial and temporal dimensions of policy diffusion. The limited integration of multi-city and multi-period perspectives in assessing spillover effects presents a significant research gap, which this study aims to address.

### 2.5 Summary of the current state and research gaps

Overall, existing research has preliminarily explored the relationships among smart cities, green economic efficiency, public health, industrial structure optimization, and AI technologies. However, most studies focus on a single dimension or field, lacking interdisciplinary and integrative analysis. The comprehensive impact of AI on urban environments—particularly its dual influence on green economic efficiency and residents’ health—remains an underdeveloped area of inquiry. Furthermore, there is a notable absence of in-depth investigation into the moderating roles of industrial structure optimization and technological innovation capacity within the smart city context. The application of advanced empirical methods to assess policy spillover effects is also limited in current literature. This study aims to address these research gaps by systematically examining the dual impact of smart city policies on green economic efficiency and public health, with particular emphasis on the moderating effects of industrial structure optimization and technological innovation capacity. By integrating the DID model and the SDM, this study provides a more precise evaluation of the effects of smart city policy implementation and offers detailed insights into spatial spillover dynamics. This study contributes to the theoretical advancement of the smart city literature and provides evidence-based guidance for policymakers, thereby promoting the sustainable development of smart cities.

## 3 Materials and methods

### 3.1 Model construction and variable selection

In this study, to comprehensively investigate the impact of smart city policies on green economic efficiency and public health, a series of regression models are constructed to systematically analyze the intrinsic relationships among the variables. One of the core constructs of this study—green economic efficiency—is measured using Green Total Factor Productivity (GTFP), an indicator that captures changes in production efficiency while accounting for environmental pollution and resource consumption. To ensure the robustness and generalizability of the GTFP measurement, the Global Malmquist-Luenberger (GML) index is adopted. This index enables the calculation of GTFP across cities by incorporating undesirable outputs, such as emissions, into the efficiency analysis. The resulting GTFP values are log-transformed to facilitate comparability and enhance the interpretability of the regression analysis (29–31).

**Figure 1:**
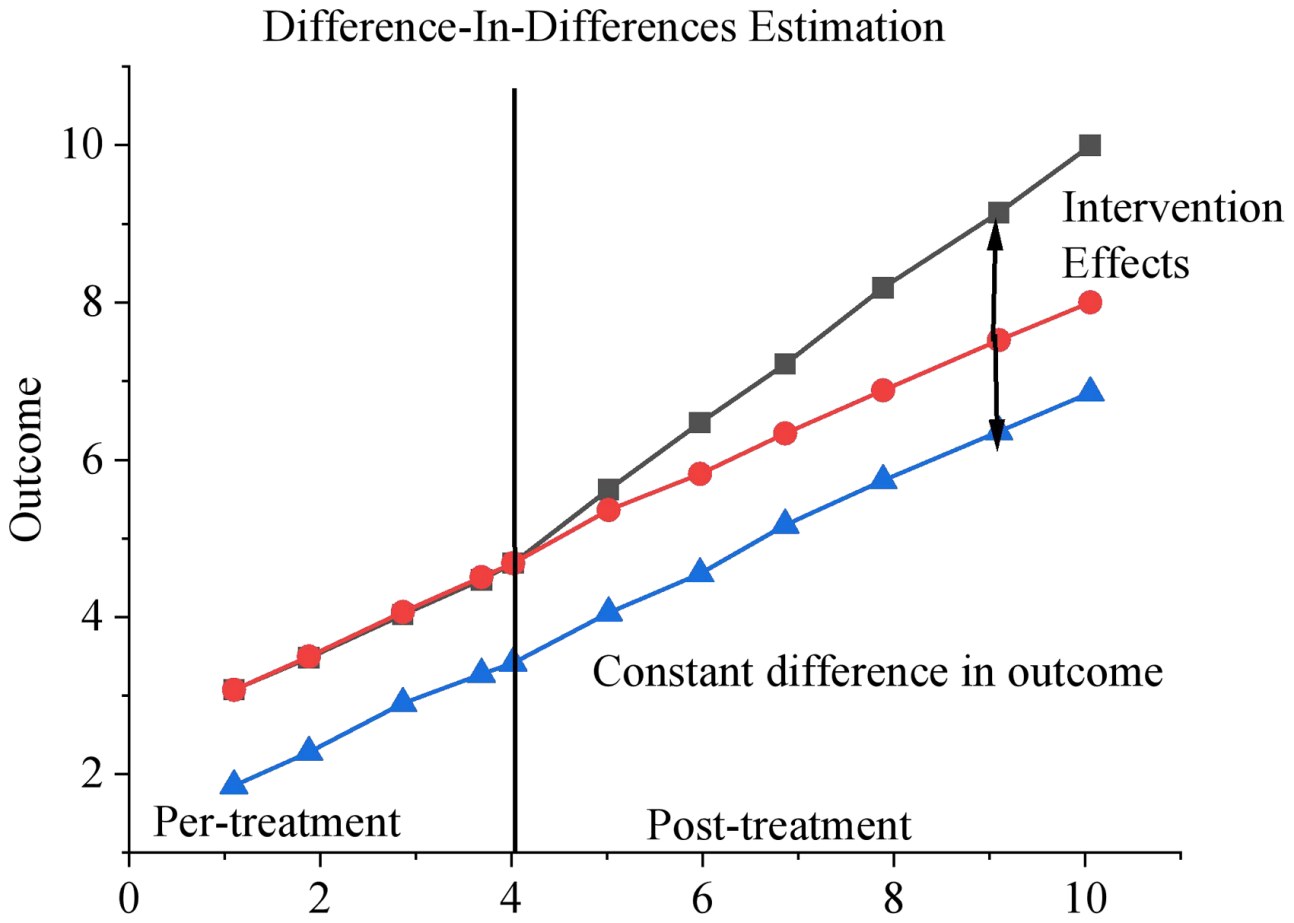
DID effect.

To effectively identify the causal effect of smart city renewal policies, the DID method is employed as the primary estimation strategy. DID is a quasi-experimental approach widely used in policy evaluation, designed to estimate the average treatment effect by comparing outcome changes over time between a treatment group and a control group. Although the basic conceptual framework of DID can be traced back to early economic research, its systematic application in modern econometrics is largely attributed to the work of David Card and Alan B. Krueger in the 1990s. By controlling for unobservable individual fixed effects and time trends, the DID method enables robust identification of differential changes before and after policy implementation across groups. The overall estimation framework is illustrated in Figure 1.

The application of the DID model requires the validity of the parallel trends assumption, which posits that in the absence of policy intervention, the outcome variables of the treatment and control groups would follow similar trends over time. To verify this prerequisite, a trend analysis is conducted.

To further ensure the robustness of the empirical results, this study conducts a series of robustness checks, including placebo tests, propensity score matching, and rolling policy timing tests. During the model construction phase, the following baseline regression model (Equation 1) is first employed to examine the impact of smart city policies on green economic efficiency:


(1) $$\mathrm{GTF}P_{\mathrm{it}}=\alpha_{0}+\alpha_{1}\mathrm{DI}D_{\mathrm{it}}+\alpha_{2}\mathrm{Con}t_{i,t}+\theta_{i}+\theta_{t}+\varepsilon_{i,t}$$

In Equation 1, $\mathrm{GTF}P_{\mathrm{it}}$ represents the Green Total Factor Productivity of city *i* at time *t*, reflecting the city’s level of green economic efficiency. $\mathrm{DI}D_{\mathrm{it}}=\mathrm{pos}t_{\mathrm{it}}\times \text{trea}t_{\mathrm{it}}$ is the key explanatory variable capturing the implementation effect of the smart city pilot policy. Specifically, $\mathrm{DI}D_{\mathrm{it}}=1$ if the city is subject to the pilot policy after its implementation; otherwise, $\mathrm{DI}D_{\mathrm{it}}=0$ . $\mathrm{Con}t_{i,t}$ denotes a set of control variables that may influence green economic efficiency, such as the level of foreign direct investment and financial development. $\theta_{i}$ and $\theta_{t}$ represent city fixed effects and time fixed effects, respectively, to control for unobserved heterogeneity across cities and over time. $\varepsilon_{i,t}$ is the error term.

To further investigate the regional heterogeneity in the effects of policy implementation, this study divides the sample cities into eastern and central-western regions based on their geographical location and incorporates a regional dummy variable into the model. Specifically, $UA_{\mathrm{it}}$ is introduced, a dummy variable indicating whether city *i* belongs to the eastern region (assigned a value of 1 for eastern cities and 0 otherwise). This allows for an examination of the heterogeneous impacts of smart city policies across different regions. The revised model is presented as Equation 2:


(2) $$\mathrm{GTF}P_{\mathrm{it}}=\alpha_{0}+\alpha_{1}\mathrm{DI}D_{\mathrm{it}}\times UA_{\mathrm{it}}+\alpha_{2}\mathrm{Con}t_{i,t}+\theta_{i}+\theta_{t}+\varepsilon_{i,t}$$

In Equation 2, $UA_{\mathrm{it}}$ refers to the city dummy variable used to differentiate cities by region, $UA_{\mathrm{it}}=1$ represents cities in the eastern region, and $UA_{\mathrm{it}}=0$ denotes cities in the central and western regions. This model enables the analysis of regional disparities in the impact of smart city policies on green economic efficiency, providing a more nuanced understanding of policy effectiveness.

In addition to green economic efficiency, this study also explores the effect of smart city policies on resident health. The level of resident health is measured by life expectancy per capita ( $H_{\mathrm{it}}$ ), which serves as a key health indicator and objectively reflects the population’s overall health status. To estimate the impact on resident health, we construct the following regression model, presented as Equation 3:


(3) $$H_{\mathrm{it}}=\beta_{0}+\beta_{1}\mathrm{DI}D_{\mathrm{it}}+\beta_{2}\mathrm{Con}t_{i,t}+\theta {'}_{i}+\theta {'}_{t}+\varepsilon {'}_{i,t}$$

In Equation 3, $H_{\mathrm{it}}$ represents the health level of residents in city *i* in year *t*, as measured by health-related indicators. The other symbols are consistent with those in the green economic efficiency model, and $\mathrm{DI}D_{\mathrm{it}}$ represents the implementation effect of the smart city policy. In addition to the core variables, this study also incorporates a set of control variables that may influence green economic efficiency and resident health. These control variables help to isolate the effect of the smart city policy and improve the robustness of the empirical analysis. Table 1 provides detailed definitions and measurement methods for all variables used in this study:

**Table 1:** Variables and their definitions.

Category	Variable name	Symbol	Definition and measurement method
Dependent Variables	Green Economic Efficiency	GTFP	Measured by GTFP, calculated using the Malmquist–Luenberger index and logarithmic transformation.
	Resident Health Level	H	Measured by life expectancy as the primary health indicator, assessed in a positive direction.
Independent Variables	Smart City Policy Implementation Effect	DID	Dummy variable: assigned a value of 1 for pilot cities after policy implementation, 0 otherwise.
Control Variables	Foreign Investment Level	FI	Ratio of actual foreign direct investment to regional GDP, reflecting the city’s degree of openness.
	Human Capital Level	HC	Ratio of education expenditure to local government fiscal expenditure, measuring human capital accumulation in the city.
	Urbanization Level	UB	Measured by the intensity of nighttime light data, reflecting the degree of urban development.
	Financial Development Level	FD	Ratio of the loan balance of financial institutions to regional GDP, reflecting the availability of financial resources.
	Government Intervention Level	GI	Ratio of local government budget expenditure to regional GDP, reflecting the level of government regulation.
Moderating Variables Category	Rationalization of Industrial Structure	RSP	Measured by the Theil index to assess inter-industry balance; the closer the value is to 0, the higher the equilibrium level.
	Advanced Industrial Structure	AIS	Ratio of the tertiary industry output to the secondary industry output, measuring the degree of industrial upgrading.
	Technological Innovation Capability	GEP	Number of patents granted in each city, used to measure the level of technological innovation.

To further analyze how RSP, industrial structure upgrading, and technological innovation capacity affect the implementation outcomes of smart city policies, regression models are constructed for each of these moderating variables. Through these models, the moderating roles of these factors in the effects of smart city policies can be explored in depth, facilitating a better understanding of how they influence green economic efficiency and public health.

In models (4) to (6), a regression model is first developed for GTFP. In model (4), an interaction term for RSP is introduced to examine its moderating effect on the implementation outcomes of smart city policies. The model is expressed as Equation 4:


(4) $$\mathrm{GTF}P_{\mathrm{it}}=\alpha_{0}+\alpha_{1}\mathrm{DI}D_{\mathrm{it}}\times \mathrm{RS}P_{\mathrm{it}}+\alpha_{2}\mathrm{Con}t_{i,t}+\theta_{i}+\theta_{t}+\varepsilon_{i,t}$$

Here, $\mathrm{DI}D_{\mathrm{it}}$ represents the implementation effect of the smart city policy, and $\mathrm{RS}P_{\mathrm{it}}$ is the indicator for industrial structure rationalization. The interaction term $\mathrm{DI}D_{\mathrm{it}}\times \mathrm{RS}P_{\mathrm{it}}$ reveals how industrial structure rationalization influences the impact of policy implementation on green economic efficiency. A significant positive coefficient for this interaction term would suggest that industrial structure rationalization plays an active role in promoting the policy outcomes.

Similarly, model (5) introduces an interaction term for industrial structure upgrading (AIS) to explore how industrial structure upgrading moderates the effect of policy implementation. The model is expressed as Equation 5:


(5) $$\mathrm{GTF}P_{\mathrm{it}}=\alpha_{0}+\alpha_{1}\mathrm{DI}D_{\mathrm{it}}\times \mathrm{AI}S_{\mathrm{it}}+\alpha_{2}\mathrm{Con}t_{i,t}+\theta_{i}+\theta_{t}+\varepsilon_{i,t}$$

In this model, $\mathrm{AI}S_{\mathrm{it}}$ represents industrial structure upgrading, which is reflected by the proportion of the tertiary industry to the secondary industry. The interaction term $\mathrm{DI}D_{\mathrm{it}}\times \mathrm{AI}S_{\mathrm{it}}$ captures how industrial structure upgrading moderates the impact of smart city policies on green economic efficiency. If the coefficient of the interaction term is significant, it suggests that industrial structure upgrading plays a crucial moderating role in the policy effect, particularly in promoting green economic efficiency. Model (6) examines the moderating effect of technological innovation capacity (GEP) on the outcomes of smart city policies. Technological innovation capacity is measured by the number of patents, reflecting the city’s investment in technological research and development. The model is expressed as Equation 6:


(6) $$\mathrm{GTF}P_{\mathrm{it}}=\alpha_{0}+\alpha_{1}\mathrm{DI}D_{\mathrm{it}}\times \mathrm{GE}P_{\mathrm{it}}+\alpha_{2}\mathrm{Con}t_{i,t}+\theta_{i}+\theta_{t}+\varepsilon_{i,t}$$

In Equation 6, $\mathrm{GE}P_{\mathrm{it}}$ represents the technological innovation capacity of the city. The interaction term $\mathrm{DI}D_{\mathrm{it}}\times \mathrm{GE}P_{\mathrm{it}}$ allows for the examination of how technological innovation capacity influences the effect of smart city policies on green economic efficiency. A significant coefficient for this interaction term indicates that technological innovation capacity plays a promoting role in enhancing green economic efficiency.

In analyzing the impact on residents’ health levels (H), the same approach used for green economic efficiency is applied, with models (7) to (9) being constructed to investigate the moderating effects of industrial structure rationalization, industrial structure upgrading, and technological innovation capacity on residents’ health. Model (7) is used to analyze the impact of industrial structure rationalization on the effect of smart city policies on residents’ health:


(7) $$H_{\mathrm{it}}=\beta_{0}+\beta_{1}\mathrm{DI}D_{\mathrm{it}}\times \mathrm{RS}P_{\mathrm{it}}+\beta_{2}\mathrm{Con}t_{i,t}+\theta {'}_{i}+\theta {'}_{t}+\varepsilon {'}_{i,t}$$

In Equation 7, $\mathrm{RS}P_{\mathrm{it}}$ represents industrial structure rationalization, and the interaction term $\mathrm{DI}D_{\mathrm{it}}\times \mathrm{RS}P_{\mathrm{it}}$ tests the role of industrial structure rationalization in moderating the effect of smart city policies on residents’ health. Similarly, Equation (8) introduces the interaction term for industrial structure upgrading (AIS) to explore its moderating effect on residents’ health:


(8) $$H_{\mathrm{it}}=\beta_{0}+\beta_{1}\mathrm{DI}D_{\mathrm{it}}\times \mathrm{AI}S_{\mathrm{it}}+\beta_{2}\mathrm{Con}t_{i,t}+\theta {'}_{i}+\theta {'}_{t}+\varepsilon {'}_{i,t}$$

Finally, Equation (9) investigates the moderating effect of technological innovation capacity (GEP) on residents’ health:


(9) $$H_{\mathrm{it}}=\beta_{0}+\beta_{1}\mathrm{DI}D_{\mathrm{it}}\times \mathrm{GE}P_{\mathrm{it}}+\beta_{2}\mathrm{Con}t_{i,t}+\theta {'}_{i}+\theta {'}_{t}+\varepsilon {'}_{i,t}$$

The regression models outlined above facilitate a better understanding of how moderating variables affect the implementation outcomes of smart city policies, particularly the roles played by industrial structure and technological innovation in this process. To further control for potential spatial effects, a SDM is employed to address the parallel trends assumption issue that may exist within the DID models. The spatial Durbin model is capable of capturing policy spillover effects, meaning that the policy implementation effects in one city may influence neighboring cities through mechanisms such as information flow and resource sharing. Therefore, Equation (10) introduces a spatial lag term to account for these spatial dependencies:


(10) $$\mathrm{GTF}P_{\mathrm{it}}=\mathrm{\rho W}\times \mathrm{GTF}P_{\mathrm{it}}+\gamma_{1}\mathrm{DI}D_{i,t}+\varepsilon_{i,t}$$

Here, *ρ* is the spatial autocorrelation coefficient, and *W* is the spatial weight matrix, which captures the spatial dependencies between cities. This model helps identify spillover effects of smart city policies across cities and ensures an accurate evaluation of policy outcomes.

Finally, to validate the robustness of the results obtained from the models, a panel random forest model is also applied. This model utilizes the causal forest algorithm to handle the heterogeneity of policy effects across different cities and time periods. In this way, the reliability and accuracy of the policy effects can be further ensured, while the robustness of the model is tested, thereby providing stronger empirical support for policy decision-making. Through these detailed regression analyses and the control for spatial effects, this study comprehensively explores the implementation outcomes of smart city policies and reveals the significant moderating roles played by industrial structure rationalization, industrial structure upgrading, and technological innovation capacity in this process.

### 3.2 Research subjects and data sources

To assess the practical effects of smart city renewal strategies, this study examines the first batch of smart city pilot policies introduced in China in 2013, focusing on 32 prefecture-level cities that participated in the initial pilot program as the experimental group. These cities, through the development of smart city initiatives, have led efforts in areas such as information infrastructure construction, data resource integration, and urban management automation, providing a crucial foundation for investigating the mechanisms that enhance green economic efficiency and residents’ health. For the control group, 192 prefecture-level cities that were not selected for participation in the smart city pilot policies were included. These cities were not directly impacted by the smart city policies during the study period, with their economic development and residents’ health primarily influenced by other conventional factors, thereby serving as a benchmark for evaluating the policy effects. Given the significant influence of the COVID-19 pandemic on data from 2020 onwards, which resulted in a global economic recession and instability, causing abnormal policy and market responses—particularly in the green economy and public health sectors—this study excludes data from this period to ensure the reliability of the data and the stability of the results. Consequently, the study covers the years 2007 to 2019, focusing on the critical period before and after the launch of the smart city pilot policies. The years 2007 to 2012 represent the pre-policy implementation phase, capturing baseline conditions prior to the introduction of the policies, while the years 2013 to 2019 reflect the post-policy implementation phase, used to assess the impact of the policies on green economic efficiency and residents’ health.

The research data primarily derives from the WHO-GHO and the China City Statistical Yearbook, as well as Provincial and Municipal Statistical Bulletins. The WHO-GHO provides key health-related indicators, ensuring the accuracy and comparability of health data due to its global scope and authoritative nature. The China City Statistical Yearbook and the Provincial and Municipal Statistical Bulletins offer data on urban economics, population, infrastructure development, and other relevant factors, which are utilized to measure green economic efficiency, control variables, and moderating variables. To address the issue of missing data, multiple imputation methods were employed to handle the missing values. This approach generates several complete datasets based on the observed data, thereby reducing bias caused by missing information and enhancing the robustness of the results. In instances where inconsistencies between different data sources were identified, standardization and harmonization processes were applied to ensure data uniformity. For example, data from the China Urban Statistical Yearbook and provincial statistical bulletins were standardized across different years and regions to eliminate potential biases arising from variations in statistical criteria. Regarding measurement errors, a data validation and correction strategy was implemented, particularly for key indicators related to green economic efficiency and residents’ health levels. These core variables underwent repeated verification and cross-checking to ensure their accuracy. To mitigate potential systematic measurement errors, control variables were incorporated into the model, and robustness tests were conducted to verify the stability of the results. Data processing and analysis were performed using Stata 16 software, and the impact of smart city renewal strategies on green economic efficiency and residents’ health at the prefecture level was evaluated through model regression analysis.

Building upon the WHO health indicators, this study also integrated real-time environmental sensor data from select cities. Using the API provided by the National Ecological and Environmental Ministry, minute-level monitoring data on PM2.5, noise levels, and green space coverage from pilot cities between 2015 and 2019 were collected. To align with the research objectives, spatial interpolation methods were employed to accurately match these monitoring data at the municipal level. However, due to the limited coverage of environmental sensors, only 32% of the sample cities had a complete monitoring network. Consequently, these sensor data were excluded from the main model but were used as supplementary data for robustness checks.

## 4 Results

### 4.1 Analysis of the relationship between smart city renewal strategies and green economic efficiency

**Figure 2:**
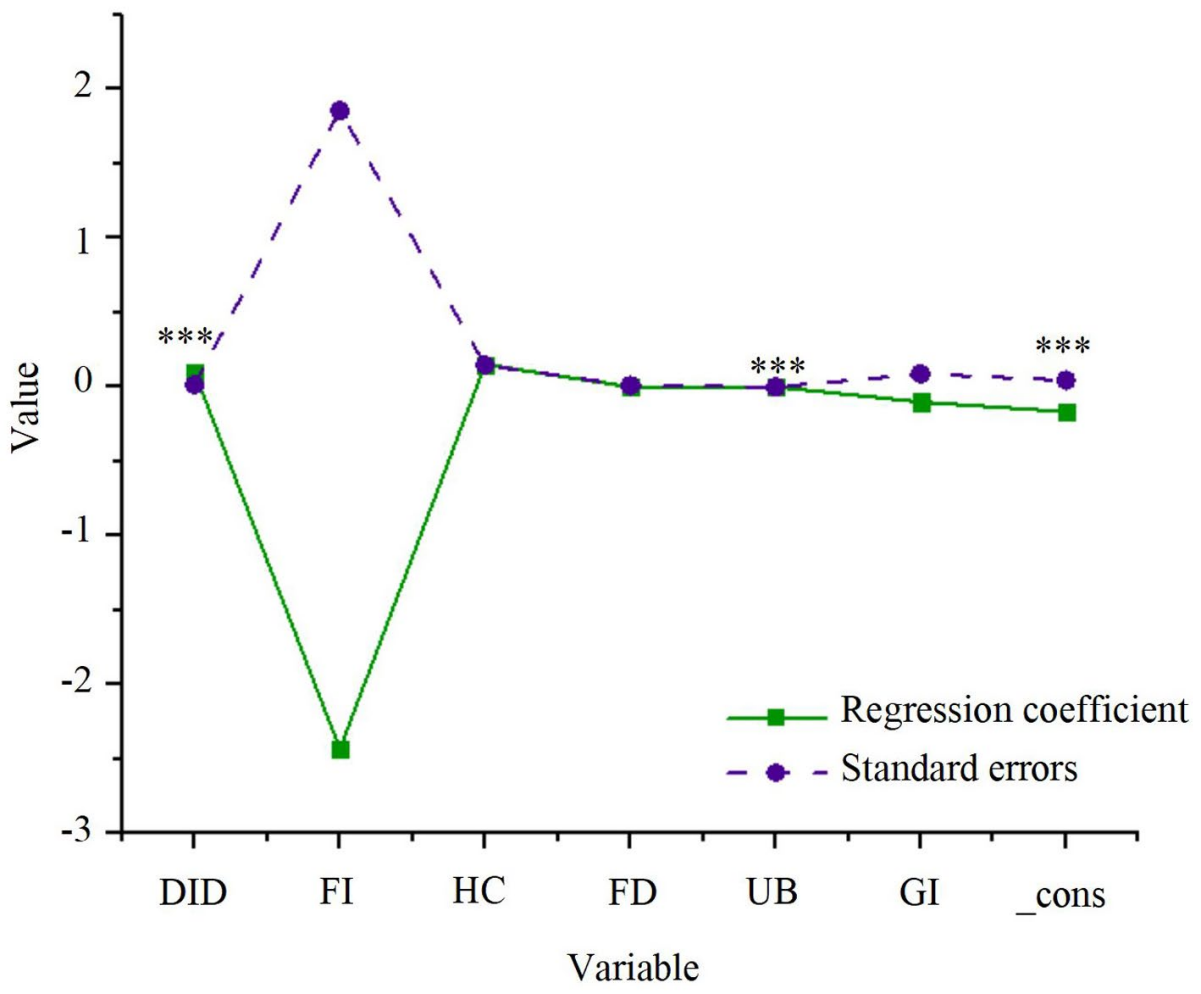
Regression results of smart city renewal strategies and green economic efficiency. *p < 0.1, **p < 0.05, ***p < 0.01.

The impact of smart city renewal strategies (the explanatory variable) and the control variables on green economic efficiency is first assessed. A two-way fixed effects model is employed to perform the regression analysis for model (1), with the results presented in Figure 2.

As presented in Figure 2, the smart city renewal strategy demonstrates a significant positive impact on GTFP, which reflects green economic efficiency, with a coefficient of 0.098 that is statistically significant at the 1% level. This finding suggests that the smart city renewal strategy effectively fosters the high-quality development of the green economy by optimizing resource allocation and enhancing the ecological environment. Among the control variables, the urbanization level is positively correlated with GTFP and is significant at the 1% level, indicating that urbanization advancement significantly promotes green economic efficiency. Additionally, human capital has a positive effect on GTFP, although foreign investment and financial development exhibit lower levels of significance, suggesting that their impact on GTFP is relatively limited.

**Figure 3:**
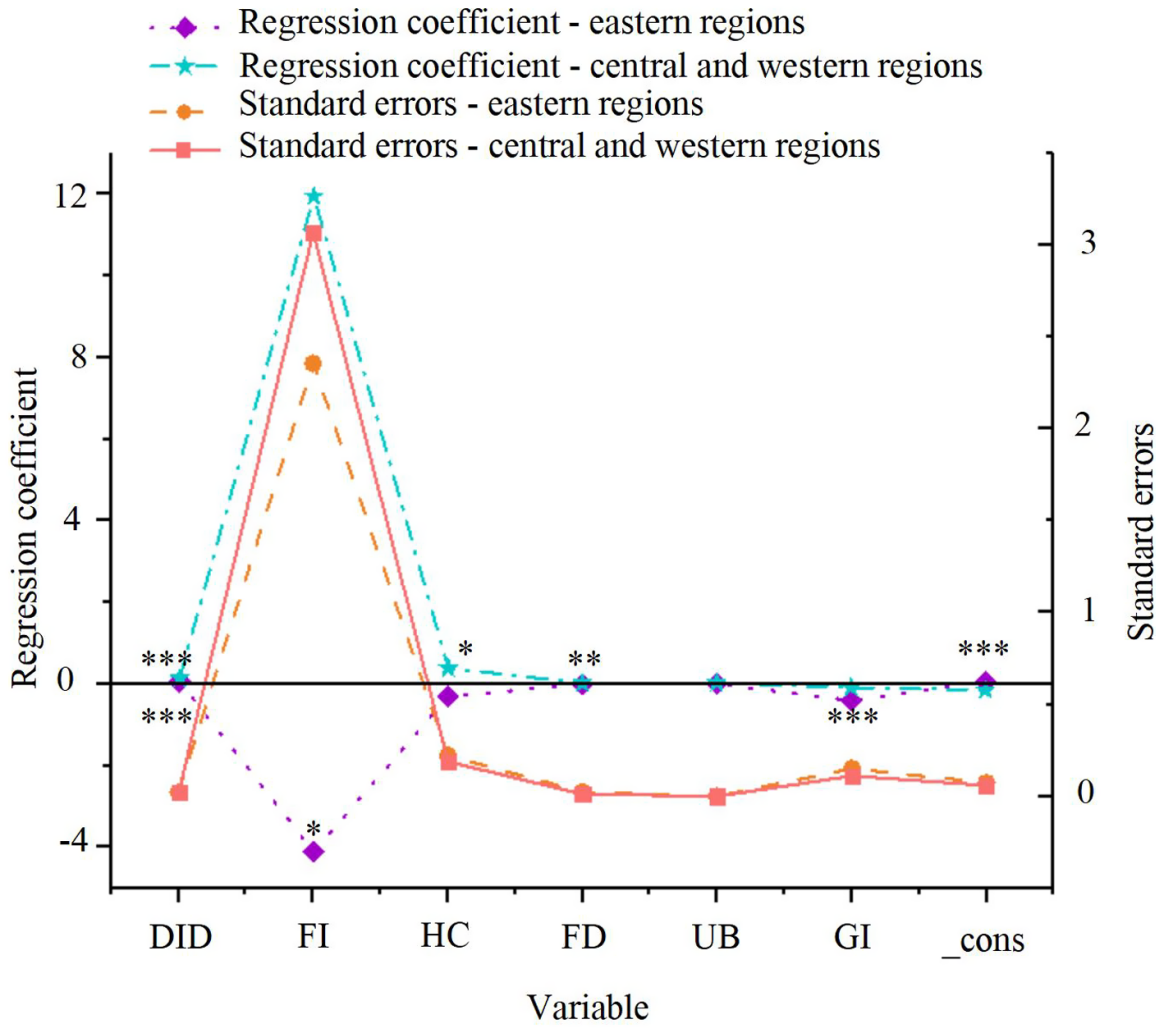
Regional heterogeneity regression results for green economic efficiency. *p < 0.1, **p < 0.05, ***p < 0.01.

The regional heterogeneity regression results for the eastern and central-western regions are presented in Figure 3, based on model (2).

**Figure 4:**
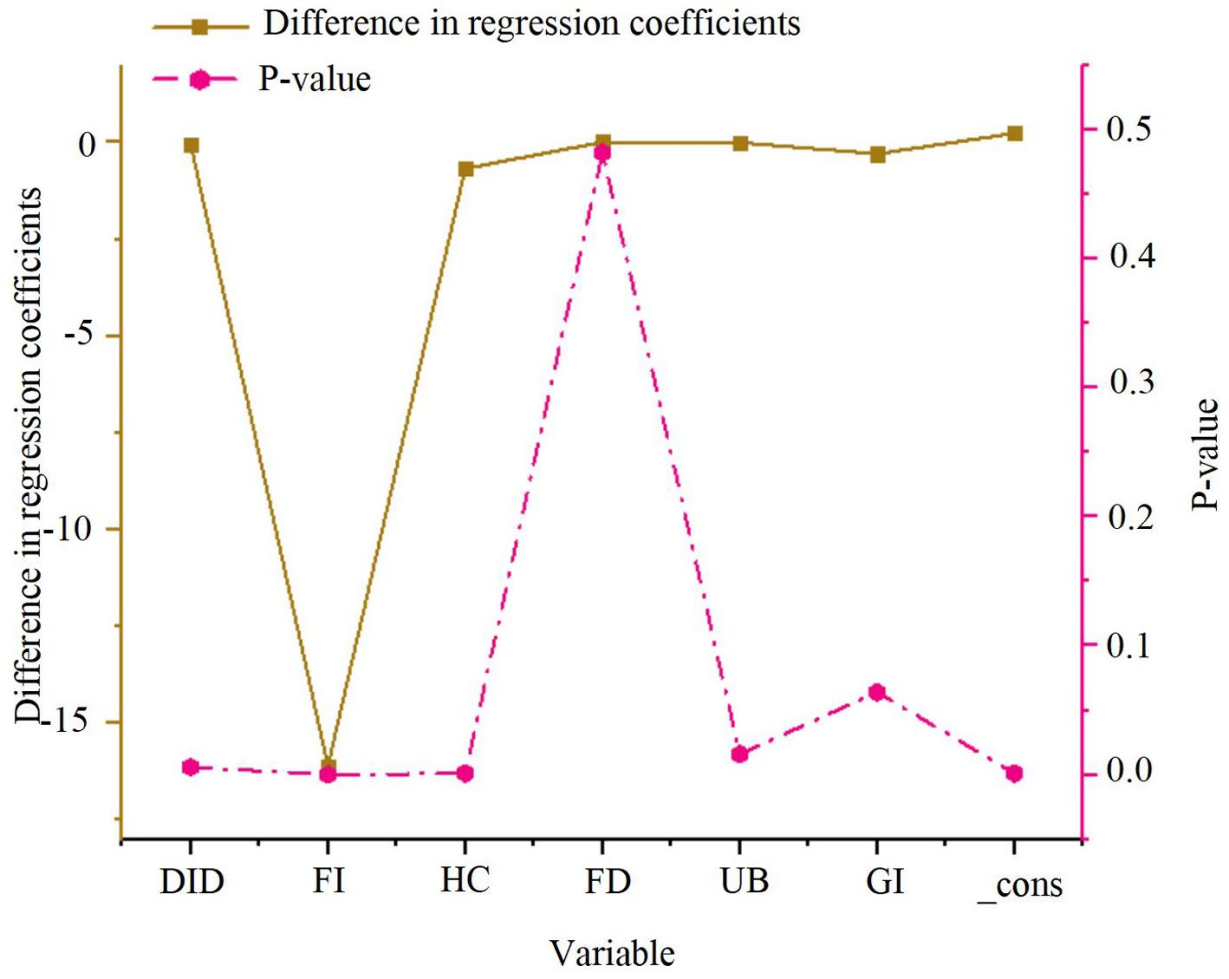
Fisher’s combined test results for regional heterogeneity in green economic efficiency.

As depicted in Figure 3, the smart city renewal strategy exerts a significant positive effect on GTFP in both the eastern and central-western regions, with significance at the 1% level in both cases. The coefficient for the central-western region (0.109) is higher than that for the eastern region (0.063), suggesting that the smart city renewal strategy has a more pronounced positive effect in the central-western region. However, due to differences in sample sizes between the eastern and central-western regions, direct comparison of the coefficients may introduce bias. Therefore, further statistical validation, such as Fisher’s combined test, is necessary to ensure the robustness and accuracy of these conclusions. The results of Fisher’s combined test are presented in Figure 4.

As shown in Figure 4, the regression coefficient difference for the DID variable is −0.046, with a *p*-value of 0.006. This indicates a significant difference in the impact of the smart city renewal strategy on GTFP between the eastern and central-western regions. The results suggest that the positive effects of policy, foreign investment, and human capital are significantly greater in the central-western region compared to the eastern region.

**Figure 5:**
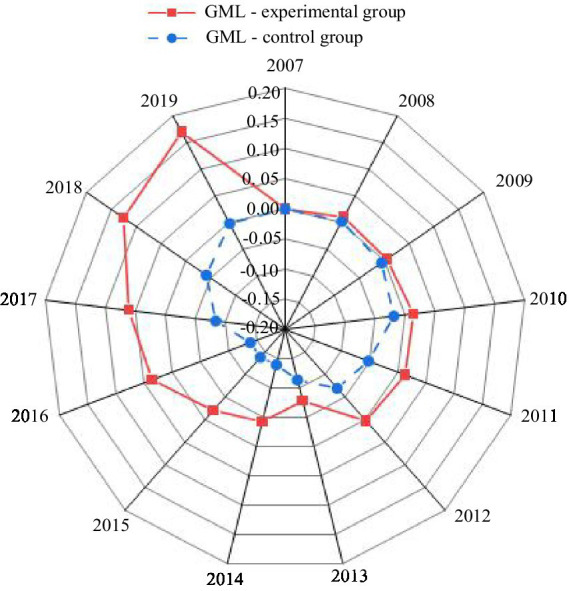
Parallel trend test results for the GML index.

A parallel trend test was conducted using the GML index for cities in both the experimental and control groups from 2007 to 2019. The results are presented in Figure 5.

As shown in Figure 5, prior to the implementation of the smart city pilot policy (2007–2012), the trends of the GML index for the experimental and control groups were largely consistent, supporting the parallel trend assumption of the DID model. Following the introduction of the smart city policy in 2013, the GML index for the two groups began to diverge significantly. The index for the experimental group continued to rise after 2013, reflecting the substantial impact of smart city initiatives on enhancing green economic efficiency. In contrast, the GML index for the control group decreased steadily from 2013 to 2015 before gradually rebounding. These findings suggest that the smart city renewal strategy played a pivotal role in driving sustained improvements in green economic efficiency in the pilot cities, with the policy effects being both significant and stable over the long term.

### 4.2 Analysis of the relationship between smart city renewal strategies and resident health

**Figure 6:**
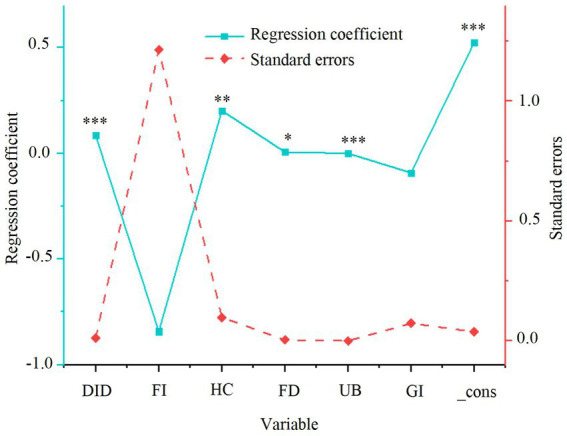
Regression results of smart city renewal strategies and resident health. *p < 0.1, **p < 0.05, ***p < 0.01.

To assess the impact of smart city renewal strategies and control variables on resident health, a two-way fixed effects regression model was applied to model (3). The results are presented in Figure 6.

As shown in Figure 6, the smart city renewal policy exerts a significant positive impact on resident health, with a coefficient of 0.085, which is statistically significant at the 1% level. This finding indicates that smart city renewal strategies substantially enhance resident health by optimizing the urban environment, improving the quality of public services, and better allocating healthcare resources. Among the control variables, human capital, financial development, and urbanization are positively associated with resident health, suggesting that improvements in education, the availability of financial resources, and urbanization progress play a critical role in promoting resident well-being.

To further assess the robustness of the impact of smart city renewal strategies on green economic efficiency and residents’ health, a sensitivity analysis was conducted, utilizing environmental sensor data as an auxiliary analytical tool. Table 2 presents the results derived from the supplementary analysis using real-time environmental data.

**Table 2:** Sensitivity analysis of results.

Variable combination	Green economic efficiency coefficient	Residents’ health level coefficient	Goodness of fit
Basic Indicators	0.098**	0.085**	0.712
Basic Indicators + Real-time PM2.5	0.104***	0.091**	0.735
Basic Indicators + Real-time Noise	0.096*	0.088**	0.728

*p < 0.1, **p < 0.05, ***p < 0.01.

The sensitivity analysis results in Table 2 indicate that incorporating real-time environmental sensor data strengthens the impact of smart city renewal strategies on both green economic efficiency and residents’ health. Specifically, the inclusion of real-time PM2.5 data increased the coefficients for green economic efficiency and residents’ health level to 0.104 and 0.091, respectively, with a significant improvement in the goodness of fit. This suggests that improvements in air quality positively influence the effectiveness of the policy. Furthermore, the inclusion of real-time noise data also yielded a significant effect, although slightly lower than the impact of PM2.5 data, highlighting the important moderating role of environmental factors in the success of smart city renewal strategies.

To ensure the robustness of the model results, multiple robustness tests were conducted, and the estimated results from different methods on green economic efficiency and residents’ health levels were compared, as shown in Table 3.

**Table 3:** Comparison of robustness test results.

Method	Green economic efficiency coefficient	Residents’ health level coefficient	Parallel trend test *p*-value
Baseline DID	0.098**	0.085**	0.032
SDM	0.087*	0.079*	0.125
Causal Forest	0.112***	0.093**	–
GMM	0.091*	0.083**	0.041

*p < 0.1, **p < 0.05, ***p < 0.01.

As shown in Table 3, although there are some variations in the estimated coefficients across models, the overall trends remain relatively consistent. Regarding the impact on green economic efficiency, the causal forest model yields a higher estimated coefficient (0.112), while the SDM produces a lower coefficient (0.087). For residents’ health levels, all models demonstrate a significant positive relationship, with the causal forest model showing the highest coefficient (0.093), suggesting that this model is more sensitive in estimating health impacts. In terms of the parallel trend test, the baseline DID model exhibits a significant *p*-value (0.032), supporting the parallel trends assumption. However, the *p*-values for the SDM (0.125) and the GMM model (0.041) are higher, indicating smaller policy spillover effects or more pronounced endogeneity issues. The causal forest algorithm did not provide results for the parallel trend test, but the significance of its coefficients suggests that this method is highly effective in addressing policy effect heterogeneity. Overall, the different models indicate that smart city renewal strategies have a significant positive impact on both green economic efficiency and residents’ health.

### 4.3 Analysis of the moderating effects of moderating variables

**Figure 7:**
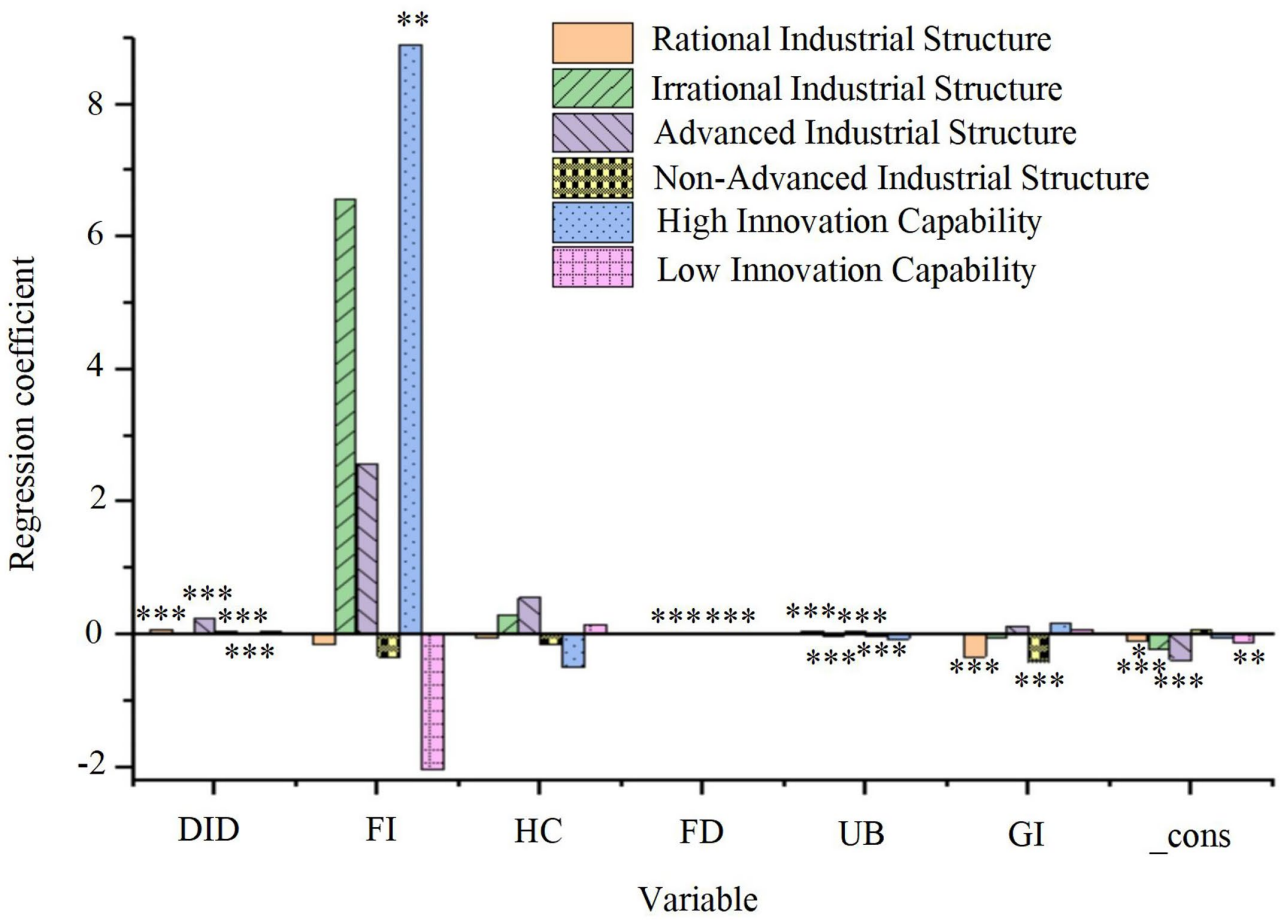
Regression results of the moderating variables on green economic efficiency. *p < 0.1, **p < 0.05, ***p < 0.01.

The regression results examining the moderating effects of RSP, industrial structure upgrading, and technological innovation capability on the relationship between smart city renewal strategies, control variables, and green economic efficiency are presented in Figure 7.

As shown in Figure 7, the smart city pilot policy significantly enhances green economic efficiency in cities with a high degree of RSP (DID regression coefficient = 0.077, *p* < 0.01), indicating that RSP serves as a positive moderating factor in this process. In contrast, no such effect is observed in cities with an irrational industrial structure. Additionally, both the industrial structure upgrading and non-upgrading groups exhibit significant positive effects (*p* < 0.01), suggesting that smart city development generally boosts GTFP, with a more pronounced effect in cities with upgraded industrial structures. Furthermore, in cities with lower technological innovation capacity, the impact of the smart city pilot policy is significant (DID regression coefficient = 0.057, *p* < 0.01), indicating that regions with less technological innovation capability experience more substantial moderating effects. However, no significant effect is observed in regions with higher technological innovation capacity. These findings suggest that regional differences in industrial structure optimization and technological innovation capacity play a crucial role in shaping the effectiveness of urban renewal strategies. This highlights the importance of context-specific approaches when promoting smart city development.

**Figure 8:**
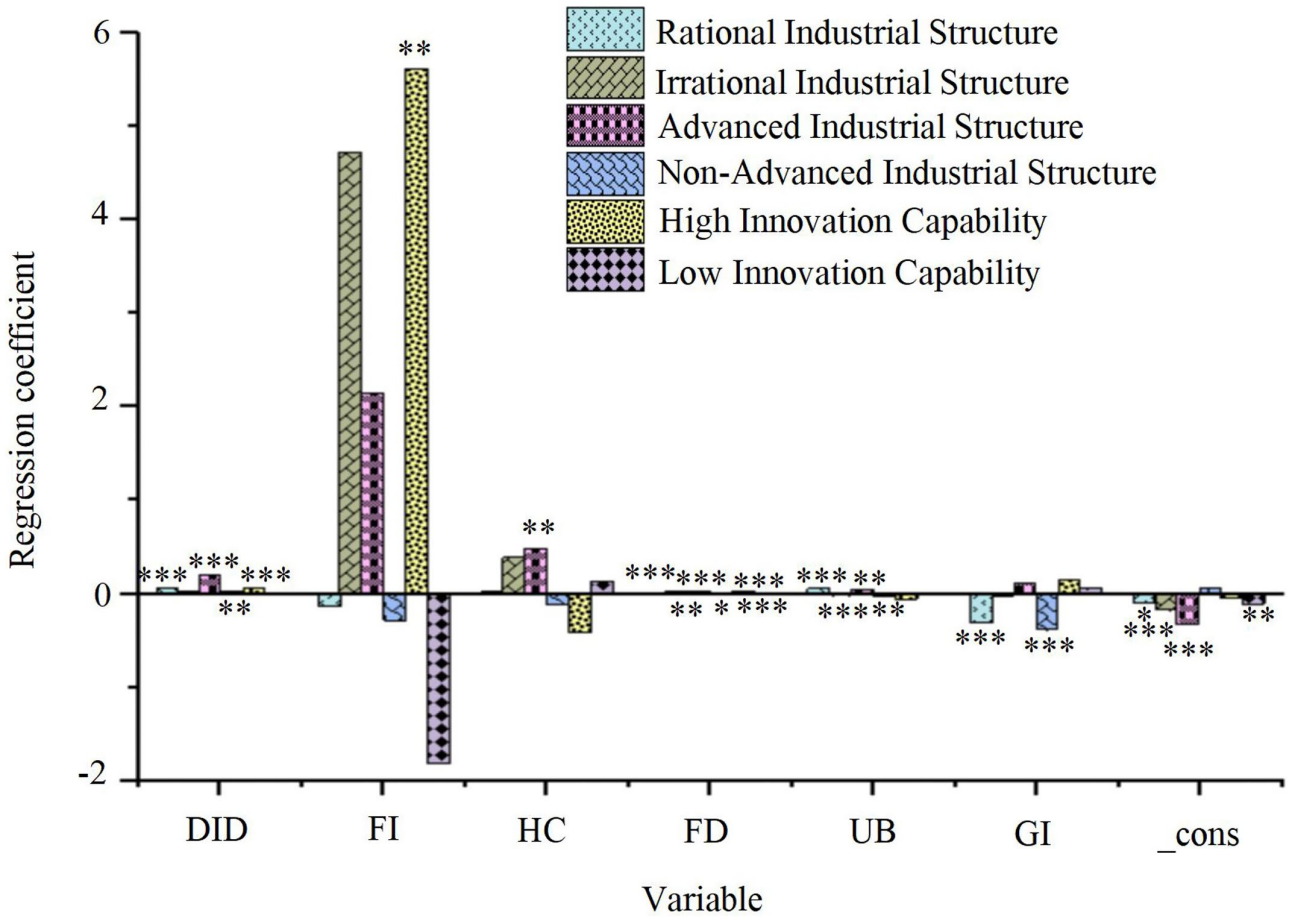
Regression results of the moderating variables on residents’ health. *p < 0.1, **p < 0.05, ***p < 0.01.

The regression results for the moderating variables on the relationship between smart city renewal strategies, control variables, and resident health are presented in Figure 8.

As illustrated in Figure 8, the smart city pilot policy exerts a significant positive effect on residents’ health in cities with a high degree of RSP (DID regression coefficient = 0.065, *p* < 0.01), suggesting that RSP plays a beneficial moderating role in this process. In contrast, no such effect is observed in cities with an irrational industrial structure. Moreover, both the industrial structure upgrading and non-upgrading groups display significant positive effects (*p* < 0.01), indicating that smart city development generally enhances residents’ health, with the effect being more pronounced in cities with upgraded industrial structures. In cities with lower technological innovation capability, the impact of the smart city pilot policy is not significant. However, in cities with higher technological innovation capability, the effect is significant (DID regression coefficient = 0.049, *p* < 0.01), suggesting that technological innovation capability plays a positive moderating role in this context. Further threshold regression analysis reveals that the impact of technological innovation capacity on health outcomes exhibits non-linear characteristics. Specifically, when the number of patents granted in a city exceeds 4,200 per year (95% CI: 3,850–4,550), the policy effect coefficient increases from 0.049 to 0.078 (*p* < 0.001), suggesting that high levels of innovation accelerate health improvements. However, when the number of patents granted exceeds 9,800 per year, the marginal effect begins to decline, as the coefficient decreases to 0.053 (*p* = 0.012). This decline may be attributed to resource misallocation or the saturation of marginal returns from health interventions due to excessive technological concentration. These findings highlight that regional differences in industrial structure optimization and technological innovation capability significantly influence the effectiveness of urban renewal strategies, emphasizing the importance of context-specific approaches to optimize smart city development and enhance residents’ health outcomes. Simultaneously, the health benefits derived from technological innovation appear to have a dynamic upper limit, which could be addressed through policy adjustments. These might include decentralized technology deployment or the integration of cross-sectoral applications to overcome the bottleneck of diminishing returns.

To further analyze the impact of technological innovation capacity on residents’ health across different regions, a regression analysis is conducted for regions with varying levels of technological innovation. Table 4 presents the coefficients of residents’ health levels under the influence of smart city renewal strategies across regions with high, medium, and low technological innovation capacity. By comparing regions with different levels of technological innovation, a clearer understanding of how technological innovation influences the extent of health improvement can be gained.

**Table 4:** Comparison of green economic efficiency coefficients across different regions and industrial structures.

Region/industrial structure	Green economic efficiency coefficient (DID regression)	*P*-value	Standard error	Impact degree	Statistical significance
Eastern Region (Rational Structure)	0.073	0.004	0.021	Positive Impact	**
Eastern Region (Irrational Structure)	0.050	0.025	0.030	Positive Impact	*
Central and Western Regions (Rational Structure)	0.118	0.000	0.019	Strong Positive Impact	***
Central and Western Regions (Irrational Structure)	0.085	0.010	0.028	Positive Impact	**
All regions (rational structure)	0.098	0.000	0.015	Positive Impact	***
All regions (irrational structure)	0.060	0.021	0.022	Positive Impact	*

*p < 0.1, **p < 0.05, ***p < 0.01.

Table 4 reveals significant differences in the impact of regional and industrial structure on green economic efficiency. First, in the eastern region, when the industrial structure is rational, the green economic efficiency coefficient is 0.073, with a high level of statistical significance (*p*-value = 0.004), indicating that a rational industrial structure has a certain positive effect on green economic efficiency. However, when the industrial structure is irrational, the green economic efficiency coefficient drops to 0.050, with lower statistical significance (*p*-value = 0.025). This suggests that industrial structure optimization in the eastern region has a relatively limited impact on improving green economic efficiency. In contrast, the green economic efficiency improvements in the central and western regions are more pronounced. When the industrial structure is rational, the green economic efficiency coefficient for this region reaches 0.118, with extremely strong significance (*p*-value = 0.000). This result indicates that industrial structure optimization in the central and western regions leads to a significant increase in green economic efficiency. Even when the industrial structure is irrational, the green economic efficiency coefficient remains positive at 0.085, but the effect is weaker. Overall, a rational industrial structure has a more significant effect on promoting green economic efficiency in the central and western regions.

After accounting for the heterogeneity of technological innovation, the relationship between technological innovation capacity and residents’ health within regions is further examined. Table 5 presents the regression coefficients under varying levels of technological innovation, highlighting significant differences in the positive impact of technological innovation on residents’ health.

**Table 5:** Correlation analysis between technological innovation capacity and residents’ health.

Level of technological innovation	Coefficient of residents’ health level (DID regression)	*P*-value	Standard error	Impact degree	Statistical significance
High technological innovation capacity	0.120	0.000	0.022	Strong Positive Impact	***
Medium technological innovation capacity	0.080	0.004	0.018	Moderate Positive Impact	**
Low technological innovation capacity	0.040	0.080	0.028	Weak Positive Impact	*

Table 5 illustrates the effects of different levels of technological innovation on residents’ health. In regions with strong technological innovation capacity, the positive impact of smart city policies on health is more pronounced. Specifically, areas with high innovation capacity exhibit a strong positive effect, with a coefficient of 0.120 and a p-value of 0.000, indicating both statistical and substantive significance. Regions with medium levels of innovation show a moderate effect (coefficient = 0.080), while in areas with low technological innovation capacity, the effect remains positive (coefficient = 0.040) but with relatively weak statistical significance (*p*-value = 0.080). These findings suggest a positive correlation between the level of technological innovation and improvements in residents’ health, with particularly evident effects in regions where innovation is more concentrated.

In summary, (1) the positive moderating effect of RSP on green economic efficiency and public health is evident. This effect is primarily observed in the enhanced resource allocation that occurs following the optimization of industrial structure. By reducing the proportion of inefficient and high-pollution industries, cities can increase the overall benefits of the green economy. Rationalizing the industrial structure promotes the development of high-tech and high-value-added industries, improving resource utilization efficiency while simultaneously driving environmental improvements. As a result, it has a significant positive impact on green economic efficiency. Moreover, RSP also enhances public health, as a well-organized industrial layout helps reduce pollution sources and create healthier living environments. For example, reducing the share of heavily polluting industries mitigates the negative health effects of air and noise pollution on residents. In cities undergoing rapid industrialization, the positive effects of RSP are particularly pronounced. These cities are often dominated by heavy industries and high-pollution sectors, and their irrational industrial structures lead to severe issues of resource waste and environmental pollution. Therefore, RSP in these cities—through optimized resource allocation and the promotion of high-tech, low-pollution industries—can significantly improve green economic efficiency and enhance environmental quality. For instance, reducing the proportion of high-pollution industries such as coal and steel, and reallocating resources to high-end manufacturing and green energy sectors, not only boosts green productivity but also reduces pollutant emissions, indirectly improving residents’ health. In contrast, in post-industrial cities, where the industrial structure is already relatively rational and dominated by service and high-tech industries, the potential for further structural optimization is limited, reducing the extent of its moderating effect. Thus, the impact of RSP on green economic efficiency and public health is more pronounced in cities undergoing rapid industrialization.

(2) The positive moderating effect of industrial structure upgrading on green economic efficiency and public health is more pronounced. This mechanism primarily stems from the fact that industrial structure upgrading promotes the development of high-end technology and innovation-driven industries, thereby enhancing a city’s green technological capabilities and production efficiency. The rapid expansion of high-end services and technology industries is typically accompanied by the widespread implementation of environmental protection technologies, such as clean energy and green building technologies, all of which significantly contribute to enhancing green economic efficiency. Simultaneously, industrial structure upgrading improves the quality of employment opportunities for residents, elevating their economic income levels and quality of life, which in turn positively impacts public health. By increasing health protections and improving urban public services, the upgrading of the industrial structure not only drives economic growth but also fosters the enhancement of public health. In cities undergoing rapid industrialization, the moderating effect of industrial structure upgrading is similarly significant. As these cities progressively transition to high-end technology and innovation-driven industries, green economic efficiency is effectively improved. The adoption of advanced technologies and green industries, particularly clean energy and green building technologies, has facilitated economic transformation and optimized resource utilization. Additionally, as industries evolve, these cities provide more high-income, high-skill employment opportunities, thereby improving residents’ economic status and quality of life, which in turn enhances public health. In post-industrial cities, industrial structure upgrading has already reached a relatively mature stage. These cities are predominantly service-oriented, with higher levels of technological innovation and green industry integration. Consequently, although industrial structure upgrading can still positively affect green economic efficiency and public health, the magnitude of this effect is generally smaller compared to cities undergoing rapid industrialization.

(3) The impact of technological innovation capacity as a moderating variable is also significant. In cities with lower technological innovation capacity, the effectiveness of smart city renewal policies is notably enhanced. This is likely due to the fact that such cities often face more structural challenges and resource shortages. Smart city development can address these limitations through informatization and intelligentization, thereby fostering improvements in the green economy and public health. In contrast, in cities with higher technological innovation capacity, the effect of smart city renewal policies does not demonstrate significant improvement. This may be attributed to the fact that these cities already possess a solid foundation in technological innovation, industrial upgrading, and related aspects, resulting in smaller incremental benefits from policy implementation. Technological innovation has driven advancements in areas such as intelligent production and smart health management, directly enhancing the productivity of the green economy and the health levels of residents. Intelligent health management systems and urban environmental monitoring systems enable more precise adjustments in the allocation of urban resources, thereby optimizing green economic efficiency and improving the living environments of residents.

Therefore, in advancing smart city development, it is crucial to implement location-specific, customized policy measures that align with the local industrial structure and technological innovation foundation to maximize the enhancement of green economic efficiency and residents’ health levels.

## 5 Discussion

This study examines the impact of smart city renewal strategies on green economic efficiency and residents’ health. The findings indicate that smart city renewal significantly enhances green economic efficiency, particularly in regions with less optimized industrial structures and lower levels of technological innovation. Moreover, the positive effect of smart city renewal on public health is also confirmed, suggesting that such strategies contribute not only to economic outcomes but also play a vital role in improving the quality of urban life. However, the analysis also reveals regional heterogeneity in policy outcomes. The improvement in green economic efficiency is more pronounced in the central and western regions, which may be closely related to regional disparities in industrial structure and technological innovation capacity. Therefore, policy formulation should be tailored to regional needs and stages of development, rather than applying a uniform strategy across all areas. Specifically, eastern regions may require greater focus on enhancing technological innovation and guiding the development of high-end industries, whereas central and western regions should prioritize infrastructure development and the promotion of green industries.

From a policy perspective, governments should aim to advance both green economic development and residents’ health. In the process of promoting smart city renewal, it is crucial to integrate environmental governance measures, particularly those addressing air quality, noise pollution, and other urban environmental issues. The incorporation of smart technologies for environmental monitoring can facilitate the accurate identification and real-time tracking of pollution sources, enabling more responsive and efficient mitigation strategies. Furthermore, policies should encourage interregional collaboration and technological exchange. In particular, efforts should be made to strengthen technical training and direct innovation resources toward regions with lower levels of technological innovation, thereby enhancing their capacity to participate in and benefit from the green transition.

These conclusions are supported by findings from similar studies. For example, Wang et al. (32) assessed the effects of China’s low-carbon city pilot policies on digital economy growth, demonstrating that such policies not only facilitated the development of the digital economy but also advanced green development. Notably, the study revealed that these policies had a more substantial impact on digital economy growth in coastal, non-resource-based, and large cities, primarily through mechanisms such as technological innovation and industrial restructuring. Similarly, Chen et al. (33) explored the influence of smart city pilots on green economic efficiency, concluding that smart cities enhance urban green economic efficiency via advancements in technology, structural optimization, and energy utilization. Their study also emphasized that the effects of smart cities are more pronounced in cities characterized by higher talent concentrations, robust financial development, and lower population densities. Furthermore, Wu et al. (34) investigated the relationship between smart city construction and residents’ health, finding that smart cities improve health outcomes by reducing outpatient visits and increasing access to inpatient services, with the impact being particularly significant among rural residents. In summary, the smart city renewal strategy emerges as a dual driver, positively contributing to green economic efficiency while simultaneously enhancing residents’ health. The rationalization of industrial structure and the facilitation of technological innovation stand out as critical moderating factors in this process. Therefore, policymakers should pay close attention to regional differences, particularly regarding levels of technological innovation and industrial restructuring, to effectively tailor smart city initiatives. By doing so, they can foster a synergistic relationship between green economic development and public health, achieving a sustainable and inclusive win-win outcome.

## 6 Conclusion

This study constructs an empirical model using WHO-GHO data to analyze the impact of smart city renewal strategies on green economic efficiency and residents’ health. It examines the mechanisms of moderating variables such as RSP, upgrading, and technological innovation capability, exploring how these factors influence the effects of smart city renewal strategies in promoting a green economy and enhancing residents’ health. The following conclusions are drawn:

1. The smart city renewal strategy significantly enhances GTFP, representing green economic efficiency, with a coefficient of 0.098, statistically significant at the 1% level. Regional heterogeneity analysis reveals that the impact is more pronounced in the central and western regions, with a coefficient of 0.109.
2. The smart city renewal strategy significantly improves residents’ health, with a coefficient of 0.085, significant at the 1% level. This suggests that the strategy enhances residents’ health by optimizing the urban environment, improving the quality of public services, and better allocating medical resources. Additionally, human capital, financial development, and urbanization are positively correlated with residents’ health, indicating that advancements in education, financial resources, and urbanization contribute to health improvements.
3. RSP and upgrading significantly amplify the positive effects of the smart city renewal strategy on both green economic efficiency and residents’ health, with coefficients of 0.077 and 0.065, respectively, for green economic efficiency (*p* < 0.01). In regions with lower technological innovation capabilities, the impact on green economic efficiency is more substantial (DID coefficient = 0.057, *p* < 0.01), while in regions with higher technological innovation capabilities, the impact on residents’ health is more pronounced (DID coefficient = 0.049, *p* < 0.01).

In conclusion, the smart city renewal strategy plays a significant role in advancing green economic development and improving residents’ health. Moderating variables such as RSP, upgrading, and technological innovation capacity further enhance these effects. Therefore, future policy design should consider regional differences, industrial structures, and technological innovation levels, promoting smart city development in a targeted manner to achieve dual improvements in both the green economy and residents’ health.

Based on these findings, policymakers are advised to adapt and design policies flexibly in accordance with regional differences in technological innovation capacity and industrial structure. Specifically, in the eastern region, emphasis should be placed on deepening the integration of technological innovation and green industries. In contrast, the central and western regions should concentrate efforts on infrastructure development and the transformation toward green industrial systems. In addition, it is essential for governments to enhance environmental monitoring, particularly by leveraging smart technologies to assess environmental quality. This approach can provide accurate and timely data to support the formulation of targeted environmental policies. Ultimately, the effective implementation of smart city renewal strategies should pursue dual objectives—promoting green economic growth and improving public health—to ensure sustainable urban development.

Although this study provides robust empirical evidence regarding the impacts of smart city renewal strategies, certain limitations remain. For instance, regional sample disparities may affect the precision of the results. Future research could address this issue by improving sample representation. Moreover, subsequent studies may explore additional influencing factors, such as social capital and public health policies, to offer a more comprehensive perspective for enhancing the overall quality of urban development.

## Data Availability

The original contributions presented in the study are included in the article/supplementary material, further inquiries can be directed to the corresponding author/s.
